# circ-Sirt1 controls NF-κB activation via sequence-specific interaction and enhancement of SIRT1 expression by binding to miR-132/212 in vascular smooth muscle cells

**DOI:** 10.1093/nar/gkz141

**Published:** 2019-03-01

**Authors:** Peng Kong, Yuan Yu, Lu Wang, Yong-Qing Dou, Xu-Hui Zhang, Yan Cui, Hai-Yue Wang, Yu-Tao Yong, Ya-Bin Liu, Hai-Juan Hu, Wei Cui, Shao-Guang Sun, Bing-Hui Li, Fan Zhang, Mei Han

**Affiliations:** 1 Department of Biochemistry and Molecular Biology, College of Basic Medicine, Key Laboratory of Medical Biotechnology of Hebei Province, Hebei Medical University, Shijiazhuang, China; 2 Department of Surgery, the Fourth Affiliated Hospital, Hebei Medical University, Shijiazhuang, China; 3 Department of Cardiovascular Medicine, the Second Hospital, Hebei Medical University, Shijiazhuang, China

## Abstract

NF-κB-mediated inflammatory phenotypic switching of vascular smooth muscle cells (VSMCs) plays a central role in atherosclerosis and neointimal formation. However, little is known about the roles of circRNAs in the regulation of NF-κB signaling. Here, we identify the involvement of circ-Sirt1 that was one of transcripts of SIRT1 host gene in VSMC inflammatory response and neointimal hyperplasia. First, in the cytoplasm, circ-Sirt1 directly interacts with and sequesters NF-κB p65 from nuclear translocation induced by TNF-α in a sequence-dependent manner. The inhibitory complex of circ-Sirt1-NF-κB p65 is not dependent on IκBα. Second, circ-Sirt1 binds to miR-132/212 that interferes with SIRT1 mRNA, and facilitates the expression of host gene SIRT1. Increased SIRT1 results in deacetylation and inactivation of the nuclear NF-κB p65. These findings illustrate that circ-Sirt1 is a novel non-coding RNA regulator of VSMC phenotype.

## INTRODUCTION

Inflammatory phenotypic switching of vascular smooth muscle cells (VSMCs) is associated with all stages of atherosclerosis and restenosis progression (1). Activation of nuclear factor kappa B (NF-κB), including the nuclear translocation and post-translational modifications, is a marker and key step of VSMC inflammatory phenotype (2). Recent studies suggest that circRNAs correlate with the NF-κB signal pathway in cancers (3), microbial infection (4) and Alzheimer's disease (5). However, the mechanism of circRNA action in fine-tune regulation of NF-κB signaling, especially in inflammatory phenotypic switching of VSMCs, was not elucidated.

Sirtuins are a family of histone deacetylases (HDAC). Mammals contain seven sirtuins (SIRT1–7). These proteins are involved in the regulation of gene transcription via epigenetic mechanisms (6). Several studies implicated SIRT1 in the inhibition of vascular inflammatory responses (7,8). Targets for SIRT1 deacetylation are key components of the intracellular inflammatory response. Notably, SIRT1 interacts with the NF-κB p65, and inhibits transcription via deacetylation of p65 in response to tumor necrosis factor alpha (TNF-α) (9). Up-regulation of SIRT1 expression may exert a beneficial vascular effect (10). Previous studies suggested that non-coding RNAs, such as long non-coding RNAs (lncRNAs) and miRNAs, generally regulated SIRT1 expression at both transcriptional and post-transcriptional levels (11). Circular RNAs (circRNAs) are a novel class of non-coding RNAs formed by back-splicing of exons, characterized by covalently closed loop structures with neither 5′ to 3′ polarity nor a polyadenylated tail. The majorities of circRNAs are highly conserved across species, and often show tissue- or development-specific expression pattern (12). They are highly stable *in vivo* compared with their linear counterparts, and are predominantly in the cytoplasm and can be sorted into exosomes (13). Previous studies have shown that circRNAs generally regulate homologous mRNA expression by acting as cytoplasmic microRNA ‘sponges’, platforms for RNA-binding proteins, or nuclear transcriptional regulators (14). Furthermore, it is becoming evident that circRNAs are involved in neuronal development, aging, cancer and cardiovascular disease (15). We searched circBase and found that the SIRT1 host gene may produce 11 circRNAs in the human genome. The functions and mechanisms of the circRNAs derived from the SIRT1 host gene in vascular diseases are not known.

In this study, we first determined that circ-Sirt1 derived from the SIRT1 gene was involved in inhibiting NF-κB activation by direct interaction and promoting SIRT1 expression through competitive binding to miR-132/212. The disruption of circ-Sirt1 may be a novel epigenetic mechanism in inflammatory phenotypic switching of VSMCs, and is a new biomarker and therapeutic target for atherosclerosis.

## MATERIALS AND METHODS

### Human arterial tissues and peripheral blood samples

Human renal artery samples were collected from 25 patients undergoing nephrectomy at the Fourth Hospital of Hebei Medical University (Shijiazhuang, China). Among them, there were 11 patients with atherosclerosis and 14 control samples from non-atherosclerosis patients.

We collected the plasma fractions of peripheral blood from 20 patients with coronary artery disease who exhibited a single coronary artery with a greater than 80% stenosis, and 20 control without clinically significant coronary artery occlusion on coronary angiography at the Second Hospital of Hebei Medical University (Shijiazhuang, China).

The Ethical Committee of Hebei Medical University approved all protocols using human samples. All patients or their relatives provided informed consent prior to their participation in the study. Supplementary Tables S1 and S2 present the details of all probands.

### Human arterial tissue culture

Arterial tissue culture was performed as described previously (16). Renal arteries from patients without atherosclerosis undergoing nephrectomy were removed and carefully denuded connective tissue. The vessels were cut into 2–4-mm rings and placed in Dulbecco's modified Eagle's medium (DMEM; Invitrogen) supplemented with 10% fetal bovine serum (FBS, Gibco), penicillin and streptomycin. Arterial rings were maintained at 37°C in a 5% CO_2_ incubator, and the medium was changed daily. Arterial rings were infected with Ad-Vector or Ad-circ-Sirt1 in low serum medium for 48 h prior to TNF-α (10 ng/ml, BD) stimulation.

### Cell culture and treatment

VSMCs were isolated from aortas of 80–100 g male Sprague-Dawley (SD) rats anesthetized intraperitoneally with urethane, and cultured in low glucose DMEM (Invitrogen) supplemented with 10%FBS (Gibco) at 37°C in a humidified atmosphere containing 5% CO_2._ The cells at passages 3–5 were used in all of the experiments, except if stated otherwise. Human arterial smooth muscle cells (HASMCs) (ScienCell, no. 6110) were cultured in Smooth Muscle Cell Medium containing apo-transferrin, insulin, fibroblast growth factor-2, insulin-like growth factor-1, hydrocortisone, and 2% foetal bovine serum (ScienCell, no. 1101). Human umbilical vein endothelial cells (HUVECs) were obtained from ATCC and maintained in Endothelial Cell Medium (ScienCell, no. 1001). Human embryonic kidney 293A cells were obtained from ATCC and cultured in high glucose DMEM (Invitrogen) containing 10% FBS. Before stimulation with TNF-α (10 ng/ml, BD), platelet derived growth factor-BB (PDGF-BB) (20 ng/ml, R&D Systems) and all-trans-retinoicacid (ATRA) (20 nM, Sigma), the VSMCs were incubated in serum free medium for 24 h.

### Balloon injury and adenoviral infection of rat carotid artery

All animal procedures conformed to the Guide for the Care and Use of Laboratory Animals published by the US National Institutes of Health (NIH Publication, 8th Edition, 2011) and was approved by the Institutional Animal Care and Use Committee of Hebei Medical University. Male Sprague-Dawley rats were obtained from the Experimental Animal Center of Hebei Medical University. Balloon denudation of the left common carotid artery of rats was performed as previously described (17). The common carotid artery of sham group was only isolated without balloon injury. For infection, after balloon injury, 20 μl of adenoviral vector-containing solution (10 × 10^10^ plaque-forming units/ml) were instilled into the vessel lumen, and the arterial segment was isolated and followed by temporary ligature of the external carotid artery for 20 min. The ligatures and catheter were then removed, the external carotid artery was ligated, and blood flow was restored.

### Hematoxylin and eosin (H&E) staining

Carotid arteries were harvested at 7, 14, 28 days after injury. The animals were euthanized by an intraperitoneal injection of ketamine (80 mg/kg)/xylazine (5 mg/kg). The left ventricle was cannulated and perfused with phosphate buffered saline (PBS) containing heparin, and then perfused and fixed with 4% paraformaldehyde in PBS under physiological pressure. The left carotid artery was then removed, further fixed for 16 hours, and paraffin-embedded without further dissection. Serial sections (5 μm thick) were obtained at 500 μm proximal to the ligation site. The cross-sectional areas of the intima and media were measured in H&E-stained sections in a blinded manner by a single observer using Image Pro Plus 6.0 software (Media Cybernetics). A mean value was determined from at least four sections for each animal. Neointima formation was determined as the ratio of the intimal area to the medial area (I/M).

### Adenovirus expression vector and plasmid constructs

Adenovirus vector encoding circ-Sirt1 (Ad-circ-Sirt1) and GFP control (Ad-Vector) were entrusted to Hanbio shanghai. The expression plasmid of circ-Sirt1 was created by the placement of rat entire circ-Sirt1 sequence into pcDNA3.1 circRNA Mini Vector (Addgene). The partial fragment of GFP was amplified by PCR and cloned into pcDNA3.1 circRNA Mini Vector as a control plasmid. The sequence of circ-Sirt1 deleted one or both of NF-κB binding regions were synthesized and inserted into pcDNA3.1 circRNA Mini Vector to overexpress mutant circ-Sirt1. Sequence of circ-Sirt1 was amplified by PCR and inserted into pGL3-promoter vector (Promega). SIRT1 gene 3′-UTR containing miR-132/212 target sites or its mutant sequences were inserted into pGL3-promoter vector. Each mutation was verified by DNA sequence analysis. The NF-κB luciferase reporter vector contained six tandem repeats of NF-κB binding site (GGGAATTTCC) as promoters upstream of the luciferase transcription start site in the vector (Beyotime).

### Small interfering RNA (siRNA) transfection

The siRNA duplexes targeting rat circ-Sirt1 (si-circ-Sirt1), 5′-UCCAAACAACCUCCUGUUGTT-3′ and 5′-CAACAGGAGGUUGUUUGGATT-3′ were obtained from GenePharma. Scrambled siRNA (si-Con) 5′-UUCUCCGAACGUGUCACGUTT-3′ and 5′-ACGUGACACGUUCGGAGAATT-3′ served as a negative control. The siRNAs were transiently transfected into VSMCs using Lipofectamine^®^ RNAiMAX Transfection Reagent (Invitrogen) according to the manufacturer's protocol.

### RNA isolation and quantitative reverse transcription-PCR (qRT-PCR)

Total RNAs of cells and tissue samples were isolated using TRIzol reagent (Life Technologies). The nuclear and cytoplasmic fractions were extracted using Minute™ Cytoplasmic and Nuclear Extraction Kit (Invent Biotechnologies). To quantify the amount of mRNA and circRNA, cDNAs were synthesized using the M-MLV First Strand Kit (Life Technologies), and quantitative PCRs were performed using SYBR Green qPCR SuperMix-UDG (Life Technologies). For microRNA, total RNA was extracted by using the QIAzol Lysis Reagent. Reverse transcription and qRT-PCR were performed with the miRNA Detection Kit by Sangon Biotech (Shanghai, China). Relative circRNA, mRNA or miRNA expression was normalized to GAPDH or U6 snRNA levels, using the 2^−ΔΔCt^ method, respectively. The sequence for each primer was listed in Supplementary Table S3. The average threshold cycle for each gene was determined from at least three independent experiments.

### Western blot and co-immunoprecipitation analysis

Protein extracts from VSMCs were quantified using a commercial reagent Bradford 1 × Dye Reagent (Bio-Rad) and normalized to 0.5 μg/μl for all samples. Protein extracts were separated by SDS-PAGE and transferred to PVDF membrane. After blocking with 5% milk in TBST, the membranes were incubated with primary antibodies against VCAM-1 (1:1000, Epitomics), ICAM-1 (1:1000, Abcam), MCP-1(1:500, Santa Cruz), SIRT1 (1:1000, Merck Millipore), NF-κB p65 (1:1000, GeneTex), Acetyl-NF-κB p65 (Lys310) (1:200, Cell Signaling Technology), GAPDH (1:1000, Cell Signaling Technology) or Lamin A/C (1:1000, Cell Signaling Technology) at 4°C overnight. After incubating with HRP-conjugated secondary antibody (1: 20 000, Abcam), the blots were visualized using GE ImageQuant™ LAS 4000 detection system. Band intensities were quantified with Image Pro Plus 6.0 software.

Lysates were precleared with Protein A/G PLUS-Agarose (Santa Cruz) to reduce nonspecific binding. The supernatants were immunoprecipitated with indicated antibodies at 4°C overnight, followed by incubation with Protein A/G PLUS-Agarose beads for 2 h. The agarose beads were then collected by centrifugation, washed with the lysis buffer, and resuspended in sample buffer. Bound proteins were resolved by SDS-PAGE followed by Western blot analysis as described above. These experiments were replicated three times.

### Fluorescence *in situ* hybridization

Cultured cells were prepared as described previously (18). The VSMCs were washed in PBS and fixed in 4% paraformaldehyde for 10 min and permeabilized overnight in 70% ethanol. Then the cells were rehydrated for 10  min in 50% formamide and 2 × SSC. For immunofluorescence, cells were blocked with 10% BSA in PBS for 2 h followed by incubation with NF-κB p65 (1:200, GeneTex) in PBS treated with DEPC at 4°C overnight. After washing three times in PBS, cells were incubated with secondary antibody (DyLight633 Conjugate, AmyJet Scientific).

For FISH, the cells was incubated using specific probes of circ-Sirt1 and miR-132/212 according to user manual of miRCURY LNATM microRNA ISH Optimization Kit (Exiqon). Hybridization was performed using fluorescence-labeled probes in hybridization buffer by incubation at 55°C for 1 h. After stringent washing with SSC buffer, cell nuclei were counterstained with DAPI (Invitrogen). Images were acquired using a Leica microscope (Leica SP5, Switzerland) and digitized with a software program LAS AF Lite.

### RNA immunoprecipitation assay (RIP)

VSMCs were washed in ice-cold PBS, lysed in lysis buffer (20 mM Tris–HCl, pH 7.0, 150 mM NaCl, 0.5% NP-40, 5 mM EDTA, with freshly added 1 mM DTT, 1 mM PMSF and 0.4 U/μl RNase inhibitor), and then incubated with 5 μg the primary antibody at 4°C for 2 h. 50 μl Protein A/G PLUS-Agarose (Santa Cruz) was added to each sample, and the mixtures were incubated at 4°C for 4 h. The pellets were washed with PBS and resuspended in 1 ml TRizol Reagent (Invitrogen). The precipitated RNA in the aqueous solution was subject to qRT-PCR analysis to demonstrate the presence of the binding products using respective primers. The experiment was replicated at least three times.

### RNA pull-down assay

RNA pull-down assays were performed as described (19). In brief, VSMCs were washed in ice-cold phosphate-buffered saline, lysed in 500 μl lysis buffer (20 mM Tris-HCl, pH 7.0, 150 mM NaCl, 0.5% NP-40, 5 mM EDTA, with freshly added 1 mM DTT, 1 mM PMSF, and 0.4 U/μl RNase inhibitor), and then incubated with 3 μg biotinylated DNA oligo probes against endogenous or ectopically expressed circ-Sirt1 at 4°C for 2 h. A total of 50 μl Dynabeads™ MyOne™ Streptavidin C1 magnetic beads (Invitrogen) were added to each binding reaction and further incubated at 4°C for 2 h. The beads were washed briefly with lysis buffer for three times. The bound proteins in the pull-down materials were analyzed by Western blot. The experiment was replicated three times.

### Oligonucleotide pull-down assay

DNA pull-down was performed essentially as described in previous (20). The oligonucleotides containing NF-κB consensus sequences biotin-5′-AGTTGAGGGGACTTTCCCAGG-3′ and biotin-5′-CCTGGGAAAGTCCCCTCAACT-3′ were used. Nuclear protein extracts (300 μg) were precleared with 20 μl Dynabeads™ MyOne™ Streptavidin C1 magnetic beads (Invitrogen) for 1 h at 4°C. After centrifugation for 2 min at 12 000 g, the supernatant was incubated with 200 pmol biotinylated double-stranded oligonucleotides and 10 μg poly (dI-dC) overnight at 4°C with gentle rocking. Then a total of 50 μl Dynabeads™ MyOne™ Streptavidin C1 magnetic beads (Invitrogen) was added, followed by a further 1 h incubation at 4°C. The protein-DNA-streptavidin-beads complex was washed four times with lysis buffer, separated on a 10% SDS-PAGE, and subjected to Western blot with different antibodies. The experiment was replicated three times.

### Chromatin immunoprecipitation assay

In brief, VSMCs were treated with 1% formaldehyde for 10 min to cross link proteins with DNA. The cross-linked chromatin was then prepared and sonicated to an average size of 400–600 bp. The samples were diluted 10-fold and then precleared with Protein A-Agarose (Santa Cruz) for 30 min at 4°C. The DNA fragments were immunoprecipitated overnight at 4°C with the NF-κB p65 (GeneTex) antibodies and normal rabbit IgG (Cell Signaling Technology). The precipitated DNA was recovered via phenol/chloroform extraction, and the NF-κB binding site was amplified by qPCR. The experiment was replicated at least three times.

### Luciferase reporter assay

Human embryonic kidney 293A cells were co-transfected with a miR132/212 mimic (GenePharma) or NC mimic combined with 400 ng luciferase reporter or an empty vector; and 293A cells were also transfected with a pcDNA3.1-circ-Sirt1 using Lipofectamine 2000 (Invitrogen) according to the manufacturer's protocol. Luciferase activity was measured using a Dual-Glo Luciferase Assay System (Promega) with a Flash and Glow (LB955, Berthold Technologies) reader 24 h after transfection. The specific target activity was expressed as the relative activity ratio of firefly luciferase to Renilla luciferase.

The NF-κB luciferase reporter vector is designed to measure the binding activity of NF-κB. It contains six tandem repeats of NF-κB binding site (GGGAATTTCC) as promoters upstream of the luciferase transcription start site in the vector. Luciferase activities were measured using a Dual Luciferase Assay Kit (Promega). Specific promoter activity was expressed as the relative activity ratio of firefly luciferase to Renilla luciferase.

Luciferase expression values were evaluated in six separate experiments. Each experiment was repeated three times.

### Statistical analysis

Data analysis was performed with Graphpad Prism 6 software (GraphPad Software, San Diego, CA, USA). Data are presented as the means ± standard error of the mean (SEM) from at least three independent experiments (n≥3), and each independent experiment was repeated three times to obtain the mean. The means derived from same treatment group were analyzed statistically. Differences between two groups were compared by Student's *t* tests. Categorical data were expressed as percentage and compared with the Fisher's exact test. For all statistical comparisons, a value of *P* < 0.05 was considered statistically significant, and denoted with one, two and three asterisks when lower than 0.05, 0.01 and 0.001, respectively.

## RESULTS

### circ-Sirt1 expression is decreased during neointimal formation, and acts as a novel biomarker of atherosclerosis

We first detected the circRNA candidates from the SIRT1 host gene in the human genome. Four circRNAs (hsa_circ_0093883, hsa_circ_0093884, hsa_circ_0093887, hsa_circ_0093888) from the SIRT1 host gene were identified in human VSMCs (Supplementary Figure S1A). The circ-Sirt1 isoform located at chr10: 69647174–69669199 in human genome (hsa_circ_0093887) was derived from the circularization of exon-2 to exon-7 of the SIRT1 gene (Supplementary Figure S1B). The primers that amplified the sequence encompassing back-splice site of circ-Sirt1 were designed, and RT-PCR verified the presence of circ-Sirt1 in rat VSMCs. The length of circ-Sirt1 was 927 nt (Supplementary Figure S1C-E). Multiple sequence alignments indicated that the sequence of human circ-Sirt1 was 93% homologous with rat (Supplementary Figure S1F).

**Figure 1.**
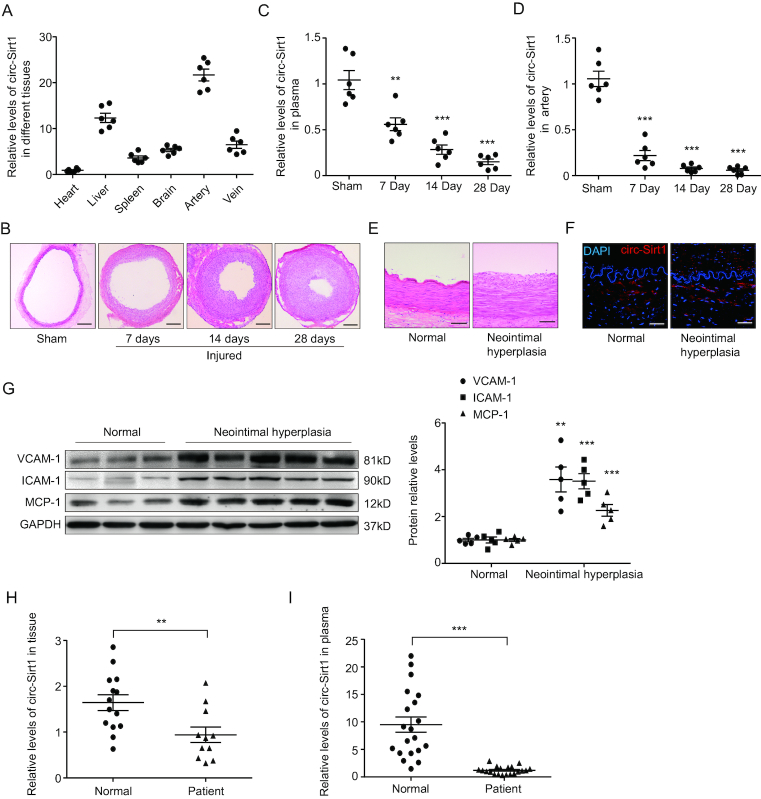
circ-Sirt1 expression is decreased during neointimal formation, and acts as a novel biomarker of atherosclerosis. (**A**) qRT-PCRs for circ-Sirt1 expression in rat different tissues. (**B**) Representative hematoxylin and eosin staining of the sections from rat carotid arteries underwent a sham operation or 7, 14 and 28 days after balloon injury. Scale bars = 200 μm. (**C** and **D**) qRT-PCRs for circ-Sirt1 expression in rat plasma (C) and carotid arteries (D) at 7, 14, 28 days after balloon injury. (**E**) Representative hematoxylin and eosin staining of cross sections from human renal arteries with neointimal hyperplasia and normal renal arteries. Scale bars = 100 μm. (**F**) RNA *in situ* hybridization for expression and location of circ-Sirt1 in normal and neointimal hyperplasia human renal arteries. Scale bars = 50 μm. (**G**) Western blot for VCAM-1, ICAM-1 and MCP-1 in human renal arteries with normal and neointimal hyperplasia renal arteries. (**H** and **I**) qRT-PCRs for circ-Sirt1 expression in normal and neointimal hyperplasia human renal arteries (H), and in plasma fraction of peripheral blood in coronary artery disease patients and control patients (I). Data are presented as mean ± SEM. *n* = 6 for A, C, D. *n* = 5 for G. n = 14 and 11 for H. *n* = 20 for I. **P< 0.01; ***P< 0.001.

We then detected the distribution of circ-Sirt1 in diverse rat tissues using qRT-PCR, and showed that circ-Sirt1 was widely expressed in several rat tissues, including heart, liver, spleen, brain, artery and vein (Figure 1A). Using a balloon injury model of rat carotid artery, we found that circ-Sirt1 levels gradually decreased in the carotid arteries and plasma at 7, 14 and 28 days after balloon injury compared to sham group (Figure 1B–D), implying that circ-Sirt1 may be involved in the vascular response to injury.

To validate the speculation, we collected renal arterial specimens from patients with or without atherosclerosis who underwent nephrectomy at the Fourth Hospital of Hebei Medical University, China. The atherosclerotic renal-artery wall exhibited neointimal hyperplasia (Figure 1E). *In situ* hybridization revealed that circ-Sirt1 expression in human renal arterial neointima was lower than normal controls (Figure 1F), accompanied with increased expression of the pro-inflammatory cytokine MCP-1 and adhesion molecules VCAM-1, ICAM-1 (Figure 1G). qRT-PCR analyses further validated these results (Figure 1H). In addition, the plasma circ-Sirt1 level was significantly decreased in patients with coronary artery disease (Figure 1I). Collectively, these results suggest that circ-Sirt1 is involved in the pathogenesis of vascular diseases, and it may act as a novel potential biomarker in the detection of patients with atherosclerosis.

### circ-Sirt1 inhibits inflammatory phenotypic switching of VSMCs

Next, we examine the expression of circ-Sirt1 in vascular cells, including human umbilical vein endothelial cells (HUVECs), human and rat VSMCs. We found that circ-Sirt1 was primarily enriched in VSMCs (Supplementary Figure S2A). The expression of circ-Sirt1 was significantly decreased in VSMCs treated with TNF-α or PDGF-BB (Supplementary Figure S2B, C), and increased in ATRA-induced VSMCs exhibiting a differentiated phenotype (Supplementary Figure S2D). We also found that circ-Sirt1 was down-regulated by 2.3-fold in exosomes secreted from PDGF-BB treated VSMCs (Supplementary Figure S2E), suggesting that circ-Sirt1 may be involved in phenotypic switching of VSMCs.

**Figure 2.**
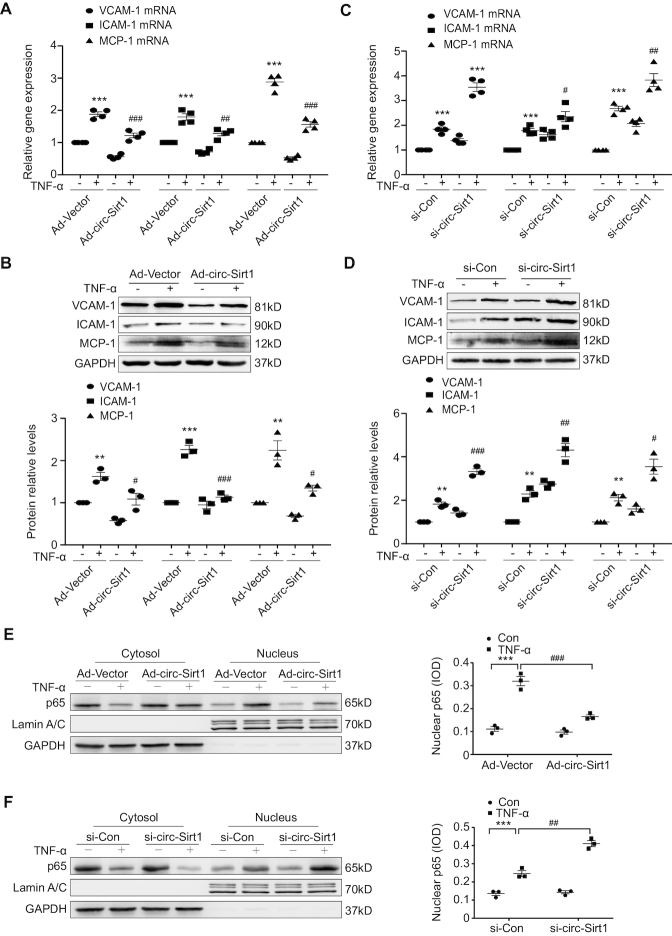
circ-Sirt1 inhibits inflammatory phenotypic switching of VSMCs. (**A** and **C**) qRT-PCRs (**B** and **D**) Western blot for VCAM-1, ICAM-1 and MCP-1 expression in VSMCs treated with TNF-α for 12 and 24 h, respectively. *P< 0.05 versus Ad-Vector+TNF-α untreated group or si-Con+TNF-α untreated group, ^#^*P*< 0.05 versus Ad-Vector+TNF-α group or si-Con+TNF-α group. (**E** and **F**) Western blot for NF-κB p65 expression in cytoplasmic and nuclear fractions of VSMCs treated with TNF-α for 30 min. Ad-Vector- or Ad-circ-Sirt1-infected VSMCs (E). si-Con- or si-circ-Sirt1-transfected VSMCs (F). Data are presented as mean ± SEM. *n* = 4 for A, C. *n* = 3 for the others. * or ^#^*P*< 0.05; ** or ^##^*P*< 0.01; *** or ^###^*P*< 0.001.

To confirm a causal relationship between circ-Sirt1 and the inflammatory response, VSMCs were treated with TNF-α following circ-Sirt1 overexpression and knockdown. We showed that exogenous circ-Sirt1 was maintained at a higher level in the presence of RNase R, which suggested the circularization of exogenous circ-Sirt1 (Supplementary Figure S3A). Overexpression of circ-Sirt1 decreased TNF-α-induced expression of VCAM-1, ICAM-1 and MCP-1 at the mRNA and protein levels in VSMCs (Supplementary Figure S3B and Figure 2A, B). Knockdown of endogenous circ-Sirt1 using a specific siRNA targeting the back-splice sequence for circ-Sirt1 which unchanged endogenous SIRT1 mRNA expression, resulted in increased expression of pro-inflammatory cytokine and adhesion molecule upon TNF-α stimulation (Supplementary Figure S3C and Figure 2C, D). NF-κB nuclear translocation is a prerequisite for the activation of pro-inflammatory gene and adhesion molecule transcription (21). We showed that TNF-α-induced nuclear translocation of NF-κB p65 was significantly inhibited in VSMCs with circ-Sirt1 overexpression, accompanied by reduced pro-inflammatory cytokine and adhesion molecule expression (Figure 2E). Conversely, knockdown of endogenous circ-sirt1 enhanced the TNF-α-induced nuclear translocation of NF-κB p65 (Figure 2F). To confirm whether double-stranded (ds) RNA-induced innate anti-viral responses influences the decrease in the transactivation potential of NF-κB, we additionally constructed the control vector pcDNA-circ-GFP, and co-transfected it with a luciferase reporter driven by a six tandem-repeat NF-κB element to HEK293A cells. We showed that none of the transfection of control vector, circ-GFP or circ-Sirt1 significantly activated NF-κB (Supplementary Figure S4A). Moreover, compared with circ-GFP-transfected VSMCs, the transfection of circ-Sirt1 decreased the TNF-α-induced production of pro-inflammatory cytokine and adhesion molecule (Supplementary Figure S4B). Taken together, these results suggest that circ-Sirt1 reduces NF-κB nuclear translocation activation, which inhibits inflammatory phenotypic switching of VSMCs.

### circ-Sirt1 interacts with and sequesters NF-κB p65 in the cytoplasm

**Figure 3.**
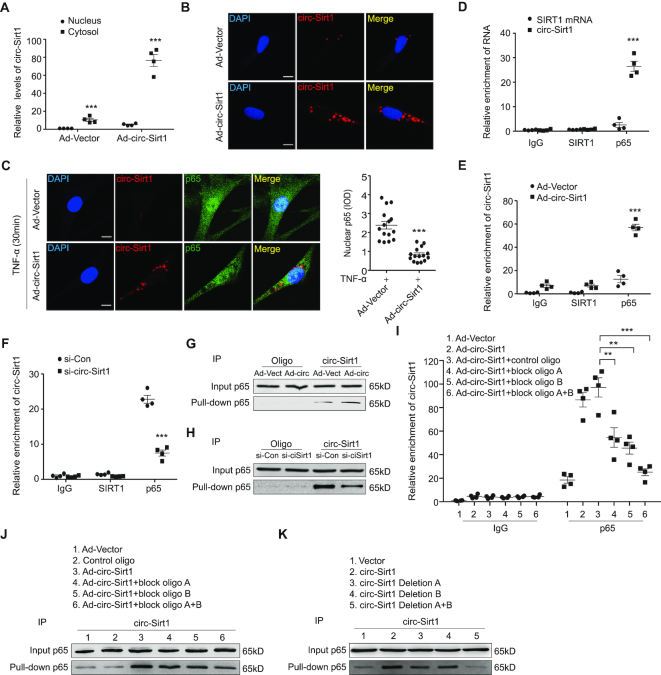
circ-Sirt1 interacts with and sequesters NF-κB p65 in the cytoplasm. (**A**) qRT-PCRs for circ-Sirt1 expression in the nuclear and cytoplasm fractions of VSMCs. (**B**) RNA *in situ* hybridization for the localization of circ-Sirt1 in VSMCs. Scale bars = 10 μm. (**C**) Overexpressing circ-Sirt1 decreased nuclear translocation of NF-κB p65 in VSMCs treated with TNF-α for 30 min. Left: Representative immunofluorescence showing the localization of NF-κB p65. Scale bars = 10 μm. Right: The quantification of nuclear NF-κB p65 without and with circ-Sirt1 overexpression. (**D–F**) RIP assay for the interactions between NF-κB p65 and circ-Sirt1 in VSMCs infected with Ad-circ-Sirt1 (E) or transfected with si-circ-Sirt1 (F). (**G** and **H**) RNA pull-down assay was performed to validate the interactions between circ-Sirt1 and NF-κB p65 in Ad-circ-Sirt1-infected VSMCs (G), and in si-circ-Sirt1-transfected VSMCs (H). (**I**) qRT-PCRs showed that antibodies against NF-κB p65 pulled down more circ-Sirt1 from the Ad-circ-Sirt1-infected VSMCs, but not in VSMCs treated with blocking oligos. (**J**) RNA pull-down assay showed that circ-Sirt1 probe pulled down more NF-κB p65 from the Ad-circ-Sirt1-infected VSMCs compared with VSMCs treated with blocking oligos. (**K**) circ-Sirt1 probe precipitated more NF-κB p65 in circ-Sirt1 overexpressed VSMCs, but not in VSMCs transfected with the circ-Sirt1 deletion mutants. Data are presented as mean ± SEM. *n* = 4 for A, D, E, F, I. *n* = 15 for C. *n* = 3 for the others. **P< 0.01; ***P< 0.001.

To elucidate the mechanism of circ-Sirt1 inhibiting the nuclear translocation of NF-κB, we detected circ-Sirt1 expression in the cytoplasmic and nuclear fractions of VSMCs using qRT-PCR. We showed that circ-sirt1 was primarily expressed in the cytoplasm (Figure 3A), and *in situ* hybridization verified this finding (Figure 3B). The circ-sirt1 in the cytoplasm was significantly increased in Ad-circ-Sirt1-infected VSMCs. Unlike circ-Sirt1, NF-κB p65 was located in the nucleus after treatment with TNF-α. However, circ-Sirt1 overexpression resulted in a cytoplasmic sequestration of NF-κB p65 under the same conditions (Figure 3C and Supplementary Figure S5A).

We further examined the potential interactions of circ-Sirt1 with NF-κB p65 in VSMCs using RNA immunoprecipitation (RIP). The mixture was precipitated using an NF-κB p65 antibody and subjected to real-time PCR using specific primers for circ-Sirt1 and linear SIRT1 mRNA. We showed that circ-Sirt1 was pulled down by antibody against NF-κB p65, but the linear SIRT1 mRNA not (Figure 3D). The interaction of circ-Sirt1 with NF-κB p65 was enhanced by circ-Sirt1 overexpression, and decreased by its knockdown (Figure 3E, F). To further confirm this interaction, lysates from Ad-Vector- and Ad-circ-Sirt1-infected VSMCs were incubated with a biotinylated DNA probe that was designed to specifically detect circ-Sirt1. The mixture was subsequently pulled down with streptavidin beads, followed by real-time PCR. We determined that the DNA probe was specific and effective for circ-Sirt1 detection (Supplementary Figure S5B, C). The NF-κB p65 pulled down by the circ-Sirt1 probe was enhanced in cells infected with Ad-circ-Sirt1 (Figure 3G), and decreased following circ-Sirt1 knockdown (Figure 3H). We also found that interactions between circ-Sirt1 and NF-κB p65 existed in human VSMCs under the same conditions (Supplementary Figure S5D–F). It has been known that endogenous IκBα exerts inhibitory effects on NF-κB p65. Therefore, we tested the potential interactions between circ-Sirt1 and IκBα. However, the circ-sirt1 probe did not precipitate IκBα protein in the RNA pull-down assay (Supplementary Figure S5G). Infection with Ad-circ-Sirt1 did not affect IκBα phosphorylation or degradation following TNF-α stimulation compared to the Ad-Vector-infected control group (Supplementary Figure S5H).

We then used computational approaches to identify the possible regions of circ-Sirt1 binding to NF-κB p65. The catRAPID program to predict protein-RNA binding (22) (http://s.tartaglialab.com/page/catrapid_group) predicted two minimal binding regions of circ-Sirt1 for the NF-κB p65 as ‘ugcaaaaagauaauaguucugacuggagcugggguuucuguuuccuguggga’ and ‘gauauuuuuaaucagguaguuccucgguguccuaggugcccagcugaugagc’ (Supplementary Figure S6A). To verify the prediction, blocking oligos that were complimentary to the NF-κB p65 binding sites in circ-Sirt1 sequence were transfected into VSMCs. We showed that the blocking oligos inihibited the interaction of circ-Sirt1 with NF-κB p65 in RIP and RNA pull-down assays without changing total circ-Sirt1 levels (Supplementary Figure S6B and Figure 3I, J). Furthermore, the interaction of NF-κB p65 with circ-Sirt1 was enhanced in VSMCs transfected with circ-Sirt1 but not the circ-Sirt1 deletion mutant (Supplementary Figure S6C and Figure 3K). These data suggest that circ-Sirt1 directly interacts with NF-κB p65 and at least partly sequesters it in the cytoplasm following TNF-α stimulation.

### circ-Sirt1 decreases TNF-α-induced NF-κB p65 acetylation and transcriptional activity via promotion of SIRT1 expression

**Figure 4.**
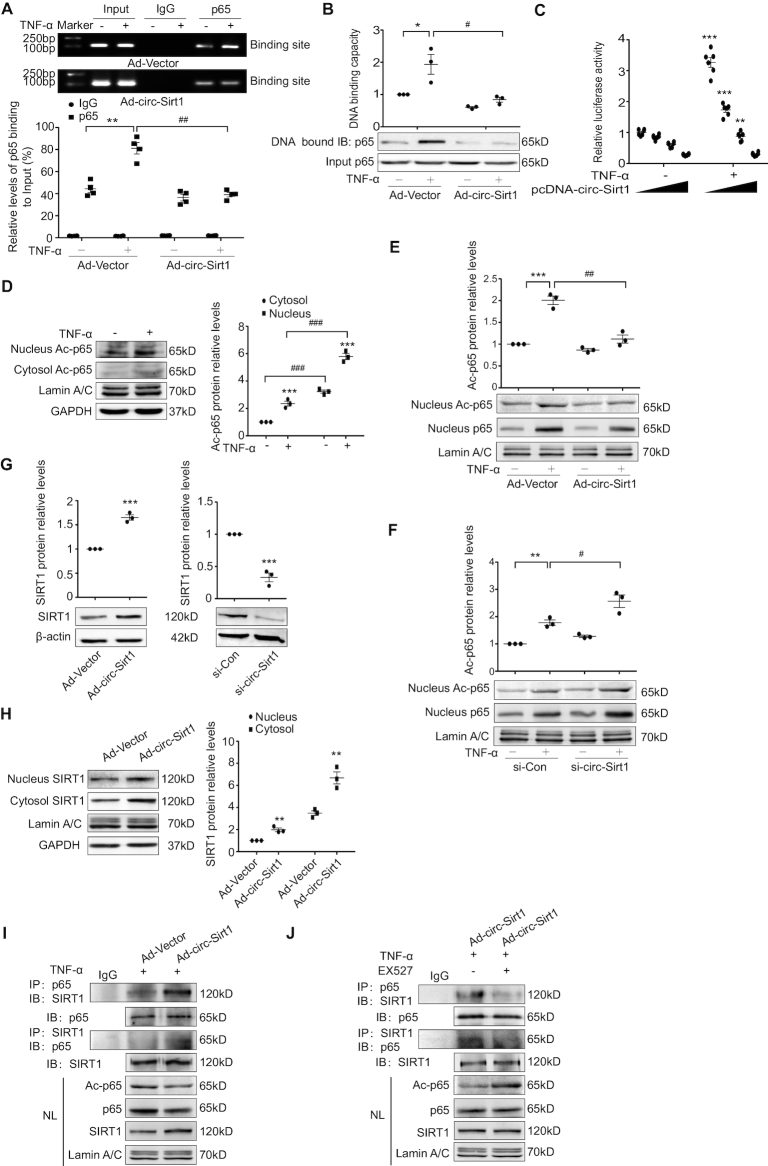
circ-Sirt1 decreases TNF-α-induced NF-κB p65 acetylation and transcriptional activity via promotion of SIRT1 expression. (**A**) ChIP assay for NF-κB p65 binding to the DNA elements in VSMCs treated with TNF-α for 1 h after infected with Ad-Vector or Ad-circ-Sirt1. (**B**) Oligonucleotide pull-down assay for the DNA-binding activity in VSMCs treated with TNF-α for 1 h after infected with Ad-Vector or Ad-circ-Sirt1. *P< 0.05 versus Ad-Vector+TNF-α untreated group, ^#^*P*< 0.05 versus Ad-Vector+TNF-α group. (**C**) Luciferase assay in HEK293A cells co-transfected with the six tandem-repeat NF-κB element and pcDNA-circ-Sirt1 followed by exposure to TNF-α for 6 h. Ratios of transfected NF-κB element and pcDNA-circ-Sirt1 were 8:1, 4:1, 2:1 and 1:1. *P< 0.05 versus TNF-α untreated group (6 × NF-κB Luc and pcDNA-circ-Sirt1 were 8:1). (**D–F**) Western blot for the expression of Ac-p65 (Lys 310) in cytoplasmic and nuclear fractions of VSMCs treated with TNF-α for 1 h (D). The expression of nuclear p65 and Ac-p65 in VSMCs treated with TNF-α for 1 h with circ-Sirt1 overexpression (E) and depletion (F). (**G** and **H**) Western blot for the expression of SIRT1. Left: In Ad-Vector- or Ad-circ-Sirt1-infected VSMCs. Right: In si-Con- or si-circ-Sirt1-transfected VSMCs. The cytoplasmic and nuclear expression of SIRT1 in Ad-Vector- or Ad-circ-Sirt1-infected VSMCs (H). (**I** and **J**) Co-IP assay for the interactions of SIRT1 with NF-κB p65 in the nuclear fraction of VSMCs treated with TNF-α for 1 h after infected with Ad-Vector or Ad-circ-Sirt1 (I), or with and without EX527 treatment (J). Data are presented as mean ± SEM. *n* = 6 for C. *n* = 3 for the others. * or ^#^*P*< 0.05; ** or ^##^*P*< 0.01; *** or ^###^*P*< 0.001.

As above mentioned, circ-Sirt1 anchored NF-κB p65 in the cytoplasm following TNF-α stimulation. However, circ-Sirt1 overexpression did not completely block the TNF-α-induced nuclear translocation of NF-κB p65 (Figures 2E, 3C and Supplementary Figure S5A). Decreasing the degree of nuclear p65 levels was less than pro-inflammatory factors (Figure 2A, B), speculating that the activity of nuclear NF-κB p65 may be weak. To validate this hypothesis, DNA binding and transcriptional activity of NF-κB were analyzed in VSMCs stimulated with TNF-α using ChIP and oligonucleotide pull-down assays, respectively. We showed that circ-Sirt1 overexpression completely abolished TNF-α-induced binding of NF-κB to the DNA elements (Figure 4A, B). The inhibitory effect of circ-Sirt1 on nuclear NF-κB activity was further supported by the reporter gene assay via transient co-expression of different ratios of circ-Sirt1 with a luciferase reporter driven by a 6 tandem-repeat NF-κB element in HEK293A cells (Figure 4C).

A previous study demonstrated that reversible acetylation regulated the duration of NF-κB transcriptional activity, and HDAC3-mediated deacetylation was an intranuclear molecular switch that controlled the NF-κB transcriptional response (23). The acetylation of NF-κB p65 Lys 310 (Ac-p65) was measured in VSMCs with circ-Sirt1 overexpression and depletion following TNF-α treatment to elucidate the mechanism underlying circ-Sirt1 decreasing nuclear NF-κB activity. We showed that TNF-α-induced Ac-p65 expression was primarily located in the nucleus (Figure 4D). Overexpression of circ-sirt1 markedly suppressed nuclear Ac-p65 expression (Figure 4E). Conversely, circ-Sirt1 silencing increased nuclear Ac-p65 expression (Figure 4F). SIRT1 is an NAD^+^-dependent protein deacetylase that mediates the deacetylation of NF-κB p65 and inhibits TNF-α-induced NF-κB activation (9). We found that the overexpression of circ-Sirt1 significantly increased SIRT1 expression at the protein levels, and knockdown of circ-Sirt1 decreased it (Figure 4G). We also observed that SIRT1 was expressed in the cytoplasm and nucleus (Figure 4H). Overexpression of circ-Sirt1 enhanced the interaction of SIRT1 with NF-κB p65 in the nucleus of VSMCs, accompanying with up-regulated SIRT1 expression and decreased NF-κB p65 acetylation, which was abolished by a specific SIRT1 inhibitor EX-527 (Figure 4I, J). Collectively, these results indicate that circ-Sirt1 enhances host gene SIRT1 expression, which contributes to decreased transcriptional activity of NF-κB p65.

### circ-Sirt1 eliminates the inhibitory effect of miR-132/212 on SIRT1 expression

**Figure 5.**
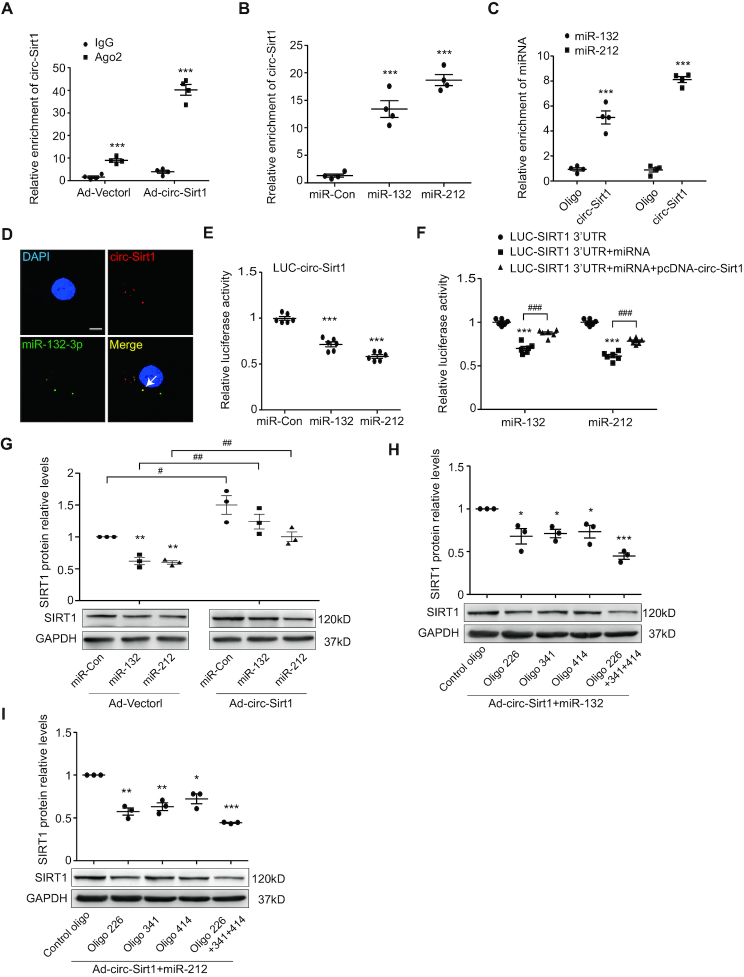
circ-Sirt1 eliminates the inhibitory effect of miR-132/212 on SIRT1 expression. (**A**) RIP assay was performed using Ago2 antibody in VSMCs. qRT-PCR was performed to detect pulled-down circ-Sirt1. (**B** and **C**) qRT-PCRs for circ-Sirt1 (B) or miR-132/212 (C) enrichment in VSMCs lysates by biotinylated-oligonucleotide probe for miR-132/212 or circ-SIRT1. (**D**) RNA *in situ* hybridization for co-localization between miR-132 and circ-Sirt1in VSMCs. Scale bars = 10 μm. (**E**) The entire circ-Sirt1 sequence was cloned into the pGL3 Luciferase Reporter to construct LUC-circ-Sirt1 vector. HEK293A cells were co-transfected LUC-circ-Sirt1 with miRNA mimics. (**F**) Luciferase reporter LUC-SIRT1 3′UTR vector and circRNAs expression vector pcDNA-circ-Sirt1 were constructed. HEK293A cells were co-transfected LUC-SIRT1 3′UTR with miRNA mimics and pcDNA-circ-Sirt1. (**G**) Western blot for the expression of SIRT1 in Ad-Vector or Ad-circ-Sirt1-infected VSMCs co-transfected with miRNA mimics. (**H** and **I**) Western blot for the expression of SIRT1 in circ-Sirt1 overexpressed VSMCs co-transfected with decoy oligos and miR-132/212. *P< 0.05 versus control oligo transfected group. Data are presented as mean ± SEM. *n* = 4 for A-C. *n* = 3 for D, G, H, I. *n* = 6 for E, F. ^#^*P*< 0.05; ** or ^##^*P*< 0.01; *** or ^###^*P*< 0.001.

circRNA acts as a miRNA sponge to regulate gene expression (24). A previous study confirmed that miR-132 and miR-212 contained identical target SIRT1 sequences (25). We used PITA (26) and RNAhybrid (27) to predict the potential target miRNAs of circ-Sirt1. We showed that circ-Sirt1 contained 3 potential interacting sites with miR-132/212 (Supplementary Figure S7A). Therefore, we detected the expression of miR-132/212 in VSMCs, and found that miR-132/212 increased in TNF-α-induced VSMCs, indicating that the miR-132/212 may be involved in VSMC inflammatory response (Supplementary Figure S7B). Ago2 protein is a core component of the RNA-induced silencing complex (RISC) that binds miRNA complexes to target mRNAs (28). We showed that circ-Sirt1 was specifically enriched in the immunoprecipitates pulled-down by the Ago2 antibody but not IgG, in the RIP assay (Figure 5A). We used a 3′ end biotin-labeled miR-132/212 to pull down circRNAs, and revealed a 13- or 18-fold enrichment of circ-Sirt1 in the pulled down sediments (Figure 5B). miR-132/212 was also enriched in circ-Sirt1-pulled down sediments (Figure 5C). The interactions between circ-Sirt1 and miR-132/212 were validated by RNA *in situ* hybridization (Figure 5D and Supplementary Figure S7C). Subsequently, We constructed a pGL3 luciferase reporter that contained the entire circ-Sirt1 sequence, LUC-circ-Sirt1, and found that miR-132/212 significantly reduced LUC-circ-Sirt1 activity by 28% and 41% in HEK293A cells (Figure 5E).

Since miR-132 and miR-212 contained the same seed sequence (29), we used TargetScan and miRanda to predict that miR-132/212 may bind the SIRT1 3′-UTR (Supplementary Figure S7D). The 3′-UTR of SIRT1 was cloned into the luciferase vector (LUC-SIRT1 3′-UTR) and co-transfected with a miR-132/212 mimic into HEK293A cells. A significant reduction in luciferase activity was observed in the presence of the miR-132/212 mimic, and mutation of the miR-132/212 target site abolished this repression (Supplementary Figure S7E). circ-Sirt1 overexpression rescued luciferase activity in LUC-SIRT1 3′-UTR and miR-132/212 mimic co-transfected HEK293A cells (Figure 5F). miR-132/212 mimic transfection significantly down-regulated SIRT1 protein expression in VSMCs. In contrast, circ-Sirt1 overexpression depressed the inhibitory effect of miR-132/212 on SIRT1 protein expression (Figure 5G). To further determine three potential miR-132/212 binding sites in circ-Sirt1, the decoy oligos complementary to 3 binding sites were synthesized and co-transfected into circ-Sirt1-overexpressed VSMCs with a 3′ end biotin-labeled miR-132/212, respectively. Using RNA pull-down assay and Western blot, we showed that the decoy oligos inhibited the circ-Sirt1 binding to miR-132/212 without affecting circ-Sirt1 level, suggesting that these three sites in circ-Sirt1 are enough to catch miR-132/212 (Supplementary Figure S7F–H and Figure 5H, I). Taken together, these data indicate that circ-Sirt1 directly binds to miR-132/212, and enhances SIRT1 mRNA activity.

### circ-Sirt1 inhibits vascular inflammation in rats and humans

**Figure 6.**
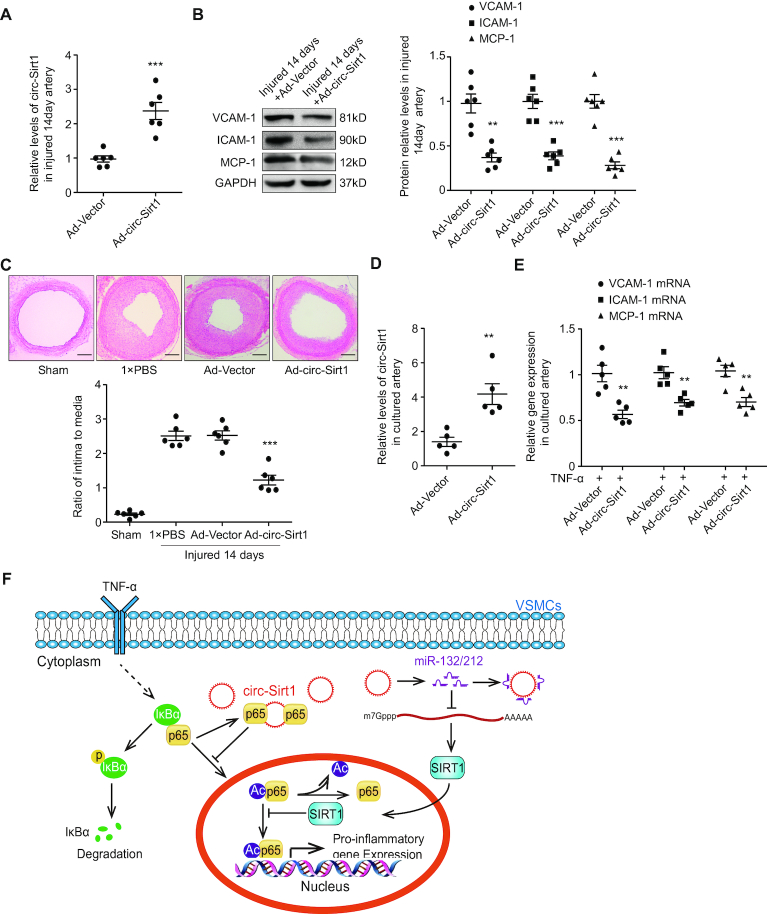
circ-Sirt1 inhibits vascular inflammation in rats and humans. (**A**) qRT-PCRs for circ-Sirt1 in carotid arteries from Ad-Vector or Ad-circ-Sirt1-infected rats carotid arteries at 14 days after balloon injury. (**B**) Western blot for the expression of VCAM-1, ICAM-1 and MCP-1 in carotid arteries from Ad-Vector or Ad-circ-Sirt1-infected rats carotid arteries at 14 days after balloon injury. (**C**) Upper: Representative hematoxylin and eosin-stained arterial sections from Ad-Vector or Ad-circ-Sirt1-infected rat carotid arteries after balloon injury. Scale bars = 200 μm. Lower: The ratio of intima to media (I/M). (**D** and **E**) qRT-PCRs for circ-Sirt1 expression (D) and the expression of VCAM-1, ICAM-1 and MCP-1 (E) in the culture of human renal artery tissues after infected with Ad-Vector or Ad-circ-Sirt1 following TNF-α treatment for 12 h. (**F**) Schematic representation of a working model by which circ-Sirt1 inhibits the activation of NF-κB through direct and indirect mechanisms, which exerts a synergistic anti-inflammatory effect. Data are presented as mean ± SEM. *n* = 6 for A–C. *n* = 5 for D, E. ** *P*< 0.01; *** *P*< 0.001.

Inflammatory responses play key roles in the initiation and progression of atherosclerosis and restenosis (1). An Ad-Vector or Ad-circ-Sirt1 was infected into rat carotid arteries after balloon injury to investigate whether circ-Sirt1 inhibits neointimal hyperplasia *in vivo*. We showed that circ-Sirt1 expression was significantly increased in carotid arteries infected with Ad-circ-Sirt1 (Figure 6A), and this increase was accompanied by a marked decrease in VCAM-1, ICAM-1 and MCP-1 expression (Figure 6B). The intima to media ratio was alleviated in Ad-circ-Sirt1-infected arteries compared to controls 14 days after balloon injury (Figure 6C). Human renal artery tissues were cultured *in vitro* and infected with Ad-circ-Sirt1 to validate the potential clinical or applied importance of circ-Sirt1. We demonstrated that exogenous circ-Sirt1 significantly reduced TNF-α-induced VCAM-1, ICAM-1 and MCP-1 mRNA level in human renal artery tissue infected with Ad-circ-Sirt1 compared to the Ad-Vector group (Figure 6D, E). These findings strongly suggest circ-Sirt1 as a potential target for the treatment of atherosclerosis and vascular diseases.

## DISCUSSION

Protein-coding exons may exhibit additional regulatory functions when expressed circRNAs in human cells (30). circRNAs generally positively regulate mRNA expression at post-transcriptional levels or exert functions as a platform for RNA-binding proteins (31,32).The present *in vitro* and *in vivo* studies identified that circ-Sirt1 produced from the SIRT1 gene was widely expressed, and it was a novel modulator of VSMC phenotype. We showed that the level of circ-Sirt1 expression was reduced in the neointimal hyperplasia of humans and rats, and VSMCs subjected to pathological stimuli. Overexpression of circ-Sirt1 was sufficient to repress inflammatory phenotypic switching of VSMCs *in vitro* and *in vivo* in humans and rats, and improved the injury-induced vascular inflammation and neointimal formation.

Phenotypic switching of VSMCs is an early event in atherosclerosis and neointimal formation. Increasing evidence suggests that multiple non-coding RNAs are involved in regulating VSMC phenotypic switching (33,34), and are becoming master regulators of inflammatory signaling to fine-tune NF-κB activity (35). The key advantage of non-coding RNA-mediated control of inflammatory programs is that they are able to provide rapid control of gene expression via mechanisms such as protein or DNA binding. Unlike miRNAs directly targeting key proteins in NF-κB signal pathway, lncRNAs carry out their roles through specific interactions with proteins, DNA and other types of RNA. Previous evidence suggested that several lncRNAs, including NKILA (36), lincRNA-p21 (37) and HOTAIR (38), regulated NF-κB signal in cancer and rheumatoid arthritis, by inhibiting IKK-induced IκB phosphorylation, interacting with p65 mRNA to inhibit translation or decreasing the level of IκB. However, little is known about the regulation and action of circRNAs in NF-κB activation and the relationship of circRNAs with VSMC phenotypic switching. The present study demonstrated that circ-Sirt1 inhibited NF-κB p65 nuclear translocation in a similar manner to IκBα, which formed a circ-Sirt1-NF-κB p65 inactive complex by sequence-specific interaction in the cytoplasm of VSMCs even if IκBα was phosphorylated and degraded after TNF-α stimulation. Furthermore, we characterized the possible binding region of circ-Sirt1 interacting with NF-κB p65, and showed that circ-Sirt1 mutants significantly inhibited the interaction of circ-Sirt1 with NF-κB p65. Our findings suggest that circ-Sirt1 is a new endogenous circRNA inhibitor of NF-κB signaling, and exhibits a synergistic effect with IκBα in VSMCs.

Although compelling evidence has indicated that some circRNAs regulate the gene expression as a miRNA sponge, only few reports show that circRNAs produced from host gene, trans-regulate the protein expression of host gene at post-transcriptional level. The covalently closed structure that increases the transcript stability, allows circRNAs to accumulate and maintain the regulatory function for a longer period of time (39). The current study found that circ-Sirt1, as a miRNA sponge for miR-132/212, promoted host gene SIRT1 expression in VSMCs. Previous study demonstrated that miR-132/212 were involved in vascular endothelial function and inflammation via targeting inhibition of SIRT1 expression in endothelial cells, contributing to impaired angiogenic responses during postnatal development and in adult mice (40,41). *In vivo*, deletion of the miR-212/132 increased endothelial angiogenic and vasodilatory functions, suggesting the vascular importance of miR-132/212 (40).We found that miR-132/212 were increased in VSMCs treated with TNF-α, and overexpression of miR-132/212 down-regulated SIRT1 expression, implying that the role of the two miRNAs in inflammatory response may be associated with reduction of SIRT1 expression, which were consistent with transcriptional regulation and cellular functions in vascular endothelial cells. In knockdown experiment, we showed that circ-Sirt1 did not change endogenous SIRT1 mRNA expression, but significantly decreased the protein level of SIRT1, suggesting the regulatory role of circ-Sirt1 at post-transcriptional level. Taken together, these findings suggest that circ-Sirt1 inhibits NF-κB activation via direct and indirect mechanisms in the cytoplasm and nucleus of VSMCs, respectively, to exert a synergistic anti-inflammatory effect.

In summary, for the first time, our study demonstrated that circ-Sirt1 exhibited beneficial protective effects against inflammatory phenotype of VSMCs. circ-Sirt1 plays dual inhibition of NF-κB activation in the cytoplasm and nucleus of VSMCs (Figure 6F). These findings reveal a critical role for circ-Sirt1 in the precise regulation of NF-κB activity, and provide novel molecular insight into the mechanisms of circRNAs in VSMC homeostasis and new therapeutic strategies for atherosclerosis and vascular diseases.

## Supplementary Material

Supplementary Data
