# Supplementary material for: circ-Sirt1 controls NF-κB activation via sequence-specific interaction and enhancement of SIRT1 expression by binding to miR-132/212 in vascular smooth muscle cells
Source: Nucleic Acids Res. 2019 Mar 1;47(7):3580–93. doi: 10.1093/nar/gkz141 (PMC6468289; doi:10.1093/nar/gkz141)
Supplement: Supplementary Data [file gkz141_supplemental_file.pdf]

SUPPLEMENTARY FIGURE LEGENDS

Supplementary Figure S1. Expression pattern of circRNA isoforms produce from SIRT1 host gene. (A) RT-PCRs for the expression of circRNA isoforms produced from SIRT1 host gene in human VSMCs. (B) Cartoon of circ-Sirt1 arises from SIRT1 host gene. (C) RT-PCR product of full-length circ-Sirt1 in rat VSMCs were analyzed and visualized by agarose gel electrophoresis. (D) RT-PCR product were purified and sequenced to confirm circ-Sirt1 junction sequences. (E) Rat circ-Sirt1 sequences. (F) circ-Sirt1 homology analysis in rat and human genome was conducted using Basic Local Alignment Search Tool (BLAST).

Supplementary Figure S2. circ-Sirt1 expression is associated with the inflammatory phenotypic switching of VSMCs. (A) qRT-PCRs for circ-Sirt1 expression in cells from different sources, including HUVECs, HASMCs and RASMCs. (B-D) qRT-PCRs for circ-Sirt1 expression in TNF-α (B), PDGF-BB (C) and ATRA (D) treated VSMCs. (E) qRT-PCRs for circ-Sirt1 expression in exosomes secreted from PDGF-BB treated VSMCs. Data are presented as mean ± SEM. n=4 for all figures. **P*<0.05; ****P*<0.001.

Supplementary Figure S3. Determination the expression of circ-Sirt1. (A) Total RNA isolated from VSMCs infected with Ad-Vector or Ad-circ-Sirt1 treated with RNase R were analyzed by qRT-PCR. The amount of circ-Sirt1 was normalized to the value measured in the mock treatment. (B and C) The expression of circ-Sirt1 increased in Ad-circ-Sirt1-infected VSMCs (B), and decreased in VSMCs treated with si-circ-Sirt1 targeting the back-splice sequence for circ-Sirt1 which unchanged endogenous SIRT1 mRNA expression (C). Data are presented as mean ± SEM. n=4 for all figures. *** P<0.001.

Supplementary Figure S4. The inhibitory effect of circ-Sirt1 on NF-κB p65 is not affected by double-stranded (ds) RNA-induced innate anti-viral responses. (A) Luciferase assay in HEK293A cells co-transfected with Vector, circ-GFP or circ-Sirt1 and the 6 tandem-repeat NF-κB element. (B) qRT-PCRs for VCAM-1, ICAM-1 and MCP-1 expression in Vector, circ-GFP or circ-Sirt1-transfected VSMCs. Data are presented as mean ± SEM values. n=6 for A. n=4 for B. * *P*<0.05; ** *P*<0.01; *** *P*<0.001.

Supplementary Figure S5. circ-Sirt1 interacts with NF-κB p65, but not IκBα. (A) Overexpressing circ-Sirt1 decreased nuclear translocation of NF-κB p65 in VSMCs treated with TNF-α for 30 min. Scale bars=50 μm. (B) The probe pulled down high levels of circ-Sirt1 in the Ad-circ-Sirt1-infected VSMCs. (C) The circ-Sirt1 probe pulled down lower levels of circ-Sirt1 in the si-circ-Sirt1-transfected VSMCs than in the control. (D) The expression of circ-Sirt1 in Ad-circ-Sirt1-infected human VSMCs. (E) RIP experiments were performed using NF-κB p65 antibody in human VSMCs. qRT-PCR for pulled-down circ-Sirt1. (F) RNA pull-down assay for the

interaction of circ-Sirt1 with NF-κB p65 in Ad-Vector or Ad-circ-Sirt1-infected human VSMCs. (G) RNA pull-down assay for the interaction of circ-Sirt1 with IκBα in VSMCs. (H) Western blot for NF-κB signaling-related proteins p-IκBα and IκBα in Ad-vector or Ad-circ-Sirt1-infected VSMCs treated with TNF-α. Data are presented as mean ± SEM. n=3 for F, G, H. n=4 for the others. * *P*<0.05; ** *P*<0.01; *** *P*<0.001.

Supplementary Figure S6. Blocking oligos and circ-Sirt1 deleted mutants inhibit the interaction of circ-Sirt1 with NF-κB p65. (A) Binding regions of circ-Sirt1 for NF-κB p65 and designed blocking oligos. (B) qRT-PCR for the expression of circ-Sirt1 in VSMCs transfected with Ad-Vector, control oligo, Ad-circ-Sirt1, Ad-circ-Sirt1 and block oligo A, Ad-circ-Sirt1 and block oligo B, Ad-circ-Sirt1 and block oligos A and B. (C) qRT-PCR for the expression of circ-Sirt1 in VSMCs transfected with Vector, circ-Sirt1, circ-Sirt1 Deletion A mutant, circ-Sirt1 Deletion B mutant, circ-Sirt1 Deletion A+B mutant. Data are presented as mean ± SEM. n=4 for B, C. *** *P*<0.001.

Supplementary Figure S7. circ-Sirt1 acts as a sponge binding miR-132/212. (A) The schematic figure showed the potential binding sites of miR-132/212 on circ-Sirt1 transcript. (B) qRT-PCR for the expression of miRNAs in TNF-α treated VSMCs for 12h. (C) RNA *in situ* hybridization for co-localization between miR-212 and circ-Sirt1 in VSMCs. Scale bars=10 μm. (D) The miR-132/212-binding sites in the 3'UTR of SIRT1 mRNA. (E) HEK293A cells were co-transfected LUC-SIRT1 3'UTR with miRNA mimics. (F) qRT-PCR for the expression of circ-Sirt1 in Ad-vector or Ad-circ-Sirt1-infected VSMCs transfected with control oligo, oligo 226, oligo 341, oligo 414 and oligo 226+341+414. (G and H) The decoy oligos inhibited the binding effect of circ-Sirt1 with miR-132/212. Data are presented as mean ± SEM. n=4 for B, F, G, H. n=6 for E. * *P*<0.05; ** *P*<0.01; *** *P*<0.001.

Supplementary Figure S1

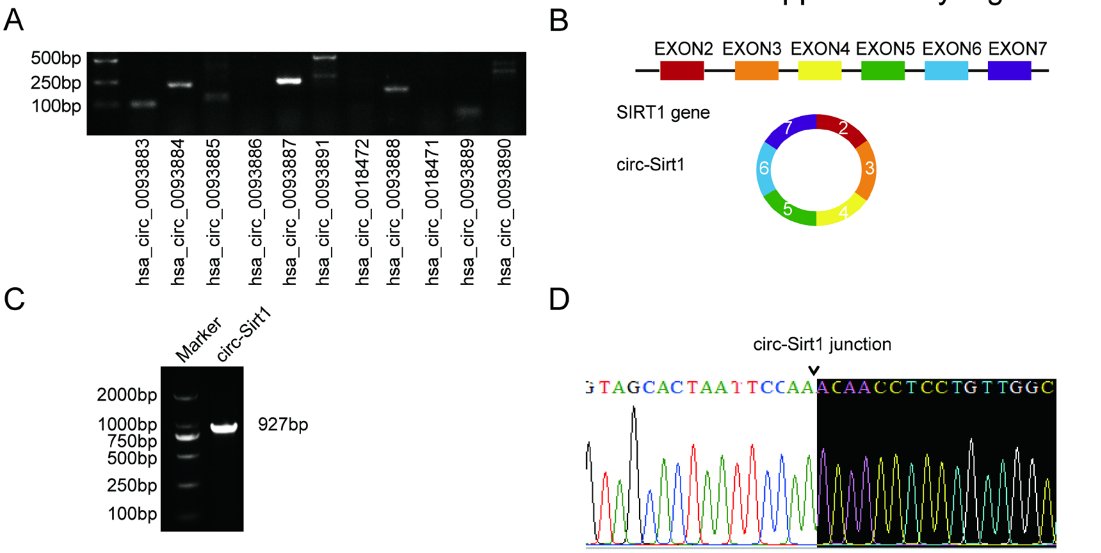

E

circ-Sirt1 sequences

ACAACCTCCTGTTGGCTGATGAGATCATCACTAATGGCTTTCATTCCTGTGAAAGT
GATGACGATGACAGAGCATCACACGCAAGCTCTAGTGACTGGACTCCAAGGCCA
CGGATAGGTCCATATACTTTTGTTCAGCAACACCTCATGATTGGCACCGATCCTCG
AACAATTCTTAAAGATTTATTACCAGAAACAATTCCTCCACCTGAGTTGGATGATAT
GACACTGTGGCAGATTGTTATTAATATCCTTTCAGAACCACCAAAGCGGAAAAAAA
GAAAAGATATTAATACAATTGAAGATGCTGTGAAGTTACTACAAGAGTGCAAAAAG
ATAATAGTTCTGACTGGAGCTGGGGTTTCTGTTTCCTGTGGGATACCTGACTTCA
GATCAAGAGATGGTATTTATGCTCGCCTTGCTGTGGACTTCCCGGATCTCCCAGA
TCCTCAAGCCATGTTCGATATTGAGTATTTTAGAAAAGACCCAAGACCATTCTTCA
AGTTTGCAAAGGAAATATATCCCGGACAGTTCCAGCCATCTCTGTGTCACAAATT
CATAGCTTTGTCAGATAAGGAAGGAAAACTACTTCGAAATTATACTCAAAATATAG
ATACCTTGGAGCAGGTTGCAGGAATCCAAAGGATCATTCAGTGTCATGGTTCCT
TTGCAACAGCATCTTGCCTGATTTGTAAATACAAAGTTGATTGTGAAGCTGTTCG
TGGAGATATTTTTAATCAGGTAGTTCCTCGGTGTCCTAGGTGCCCAGCTGATGAG
CCACTTGCCATCATGAAGCCAGAGATTGTCTTCTTTGGTGAAAACTTACCAGAAC
AGTTTCATAGAGCCATGAAGTATGACAAAGATGAAGTTGACCTCCTCATTGTTATT
GGGTCTTCTCTGAAAGTAAGACCAGTAGCACTAATTCCAA

F

Score	Expect	Identities	Gaps	Strand
1349bits(730)	0.0	860/925(93%)	0/925(0%)	Plus/Plus

```
Rat     Query  3     AACCTCCTGTTGGCTGATGAGATCATCACTAATGGCTTTCATTCCTGTGAAAGTGATGAC  62
Human   Sbjct  486   AACCTTCTGTTCGGTGATGAAATTATCACTAATGGTTTTCATTCCTGTGAAAGTGATGAG  545

Rat     Query  63    GATGACAGAGCATCACACGCAAGCTCTAGTGACTGGACTCCAAGGCCACGGATAGGTCCA  122
Human   Sbjct  546   GAGGATAGAGCCTCACATGCAAGCTCTAGTGACTGGACTCCAAGGCCACGGATAGGTCCA  605

Rat     Query  123   TATACTTTTGTTCAGCAACACCTCATGATTGGCACCGATCCTCGAACAATTCTTAAAGAT  182
Human   Sbjct  606   TATACTTTTGTTCAGCAACATCTTATGATTGGCACAGATCCTCGAACAATTCTTAAAGAT  665

Rat     Query  183   TTATTACCAGAAACAATTCCTCCACCTGAGTTGGATGATATGACACTGTGGCAGATTGTT  242
Human   Sbjct  666   TTATTGCCGGAAACAATACCTCCACCTGAGTTGGATGATATGACACTGTGGCAGATTGTT  725

Rat     Query  243   ATTAATATCCTTTCAGAACCACCAAAGCGGaaaaaaaGAAAAGATATTAATACAATTGAA  302
Human   Sbjct  726   ATTAATATCCTTTCAGAACCACCAAAAAGGAAAAAAAGAAAAGATATTAATACAATTGAA  785

Rat     Query  303   GATGCTGTGAAGTTACTACAAGAGTGCAAAAAGATAATAGTTCTGACTGGAGCTGGGGTT  362
Human   Sbjct  786   GATGCTGTGAAATTACTGCAAGAGTGCAAAAAAATTATAGTTCTAACTGGAGCTGGGGTG  845

Rat     Query  363   TCTGTTTCCTGTGGGATACCTGACTTCAGATCAAGAGATGGTATTTATGCTCGCCTTGCT  422
Human   Sbjct  846   TCTGTTTCATGTGGAATACCTGACTTCAGGTCAAGGGATGGTATTTATGCTCGCCTTGCT  905

Rat     Query  423   GTGGACTTCCCGGATCTCCCAGATCCTCAAGCCATGTTCGATATTGAGTATTTTAGAAAA  482
Human   Sbjct  906   GTAGACTTCCCAGATCTTCCAGATCCTCAAGCGATGTTTGATATTGAATATTTCAGAAAA  965

Rat     Query  483   GACCCAAGACCATTCTTCAAGTTTGCAAAGGAAATATATCCCGGACAGTTCCAGCCATCT  542
Human   Sbjct  966   GATCCAAGACCATTCTTCAAGTTTGCAAAGGAAATATATCCTGGACAATTCCAGCCATCT  1025

Rat     Query  543   CTGTGTCACAAATTCATAGCTTTGTCAGATAAGGAAGGAAAACTACTTCGAAATTATACT  602
Human   Sbjct  1026  CTCTGTCACAAATTCATAGCCTTGTCAGATAAGGAAGGAAAACTACTTCGCAACTATACC  1085

Rat     Query  603   CAAAATATAGATACCTTGGAGCAGGTTGCAGGAATCCAAAGGATCATTCAGTGTCATGGT  662
Human   Sbjct  1086  CAGAACATAGACACGCTGGAACAGGTTGCGGGAATCCAAAGGATAATTCAGTGTCATGGT  1145

Rat     Query  663   TCCTTTGCAACAGCATCTTGCCTGATTTGTAAATACAAAGTTGATTGTGAAGCTGTTCGT  722
Human   Sbjct  1146  TCCTTTGCAACAGCATCTTGCCTGATTTGTAAATACAAAGTTGACTGTGAAGCTGTACGA  1205

Rat     Query  723   GGAGATATTTTTAATCAGGTAGTTCCTCGGTGTCCTAGGTGCCCAGCTGATGAGCCACTT  782
Human   Sbjct  1206  GGAGATATTTTTAATCAGGTAGTTCCTCGATGTCCTAGGTGCCCAGCTGATGAACCGCTT  1265

Rat     Query  783   GCCATCATGAAGCCAGAGATTGTCTTCTTTGGTGAAAACTTACCAGAACAGTTTCATAGA  842
Human   Sbjct  1266  GCTATCATGAAACCAGAGATTGTGTTTTTTGGTGAAAATTTACCAGAACAGTTTCATAGA  1325

Rat     Query  843   GCCATGAAGTATGACAAAGATGAAGTTGACCTCCTCATTGTTATTGGGTCTTCTCTGAAA  902
Human   Sbjct  1326  GCCATGAAGTATGACAAAGATGAAGTTGACCTCCTCATTGTTATTGGGTCTTCCCTCAAA  1385

Rat     Query  903   GTAAGACCAGTAGCACTAATTCCAA  927
Human   Sbjct  1386  GTAAGACCAGTAGCACTAATTCCAA  1410
```

Supplementary Figure S2

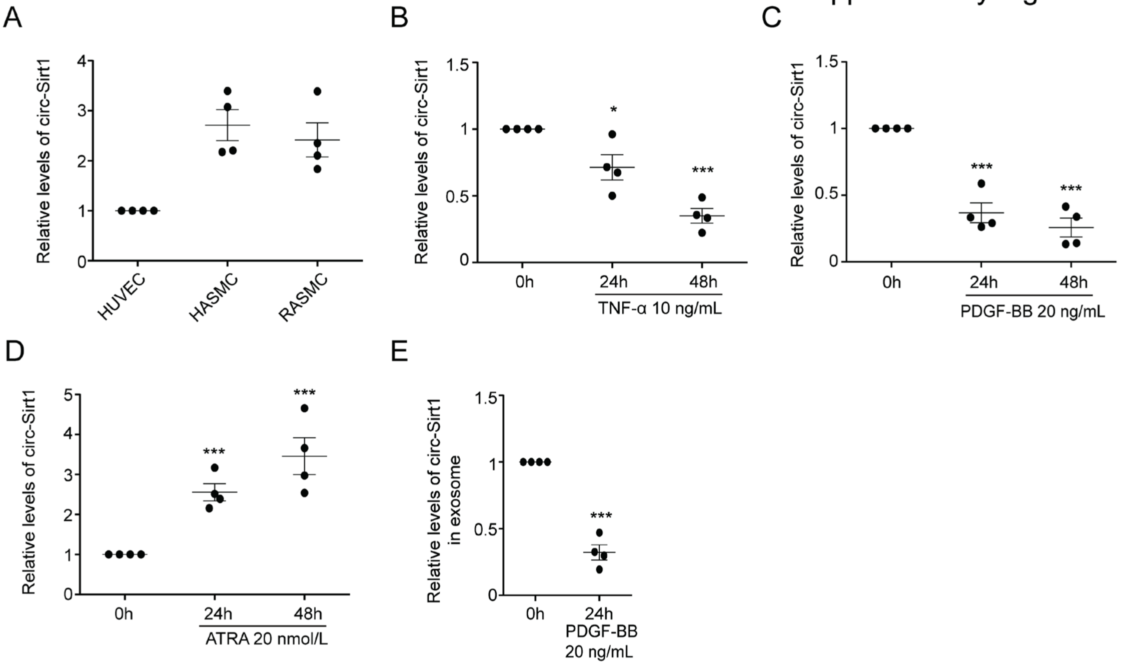

Supplementary Figure S3

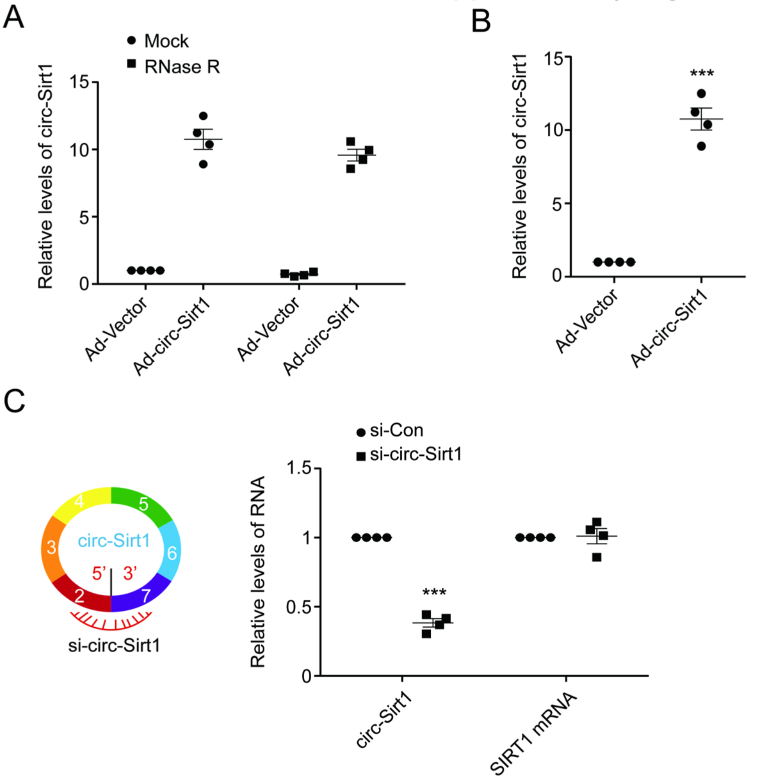

Supplementary Figure S4

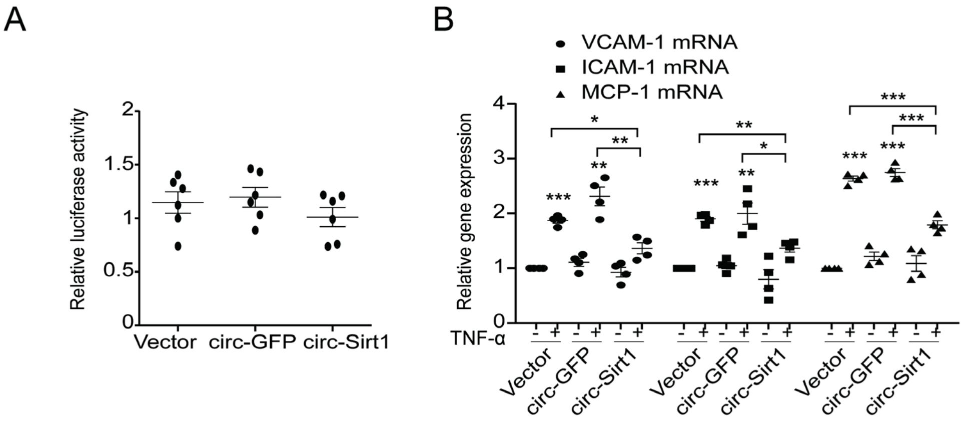

Supplementary Figure S5

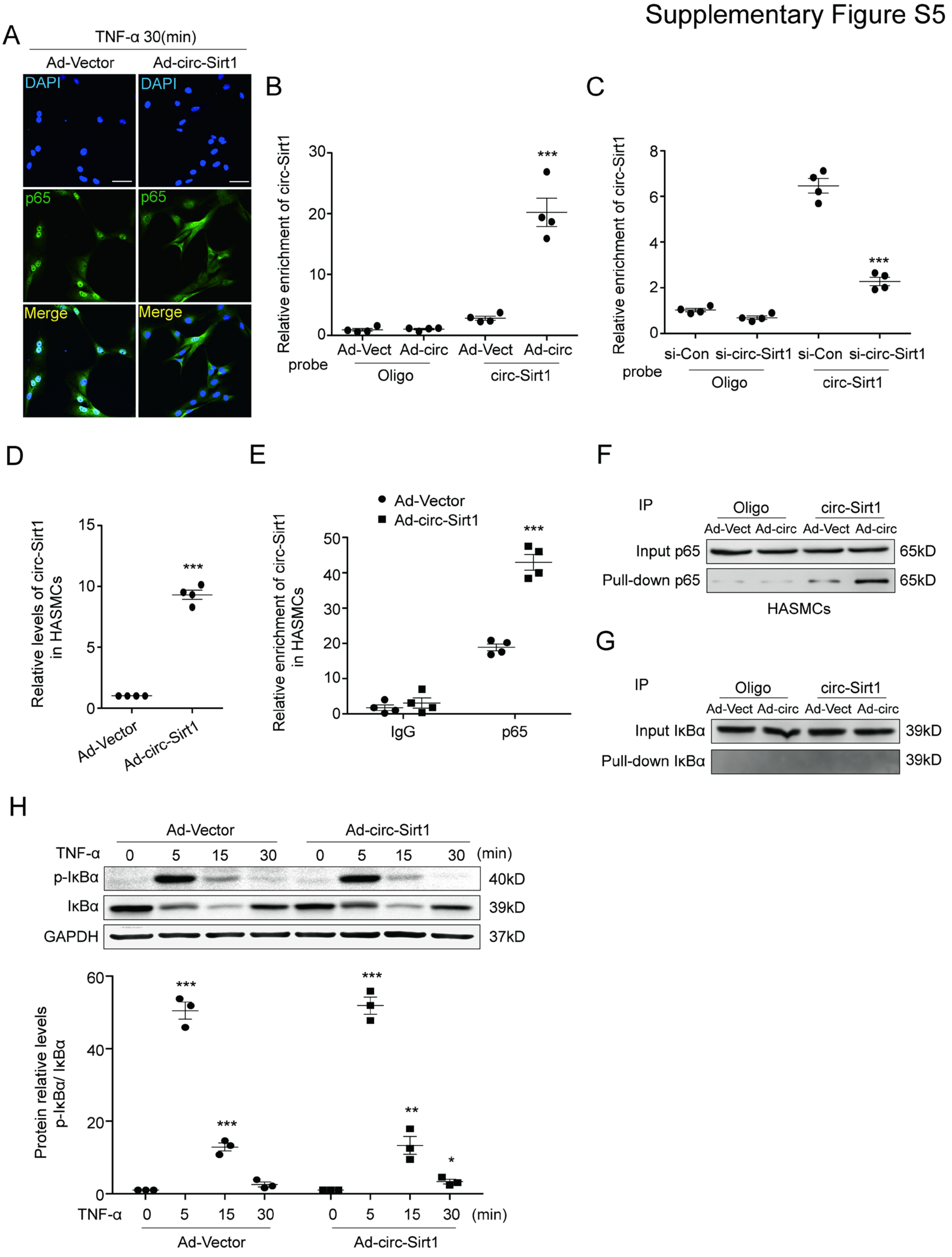

Supplementary Figure S6

A

circc-Sirt1 NF-κB p65 binding sites

5’-AAGAUGCUGUGAAGUUACUACAAGAG**UGCAAA**
AAGAUAAUAGUUCUGACUGGAGCUGGGGUUUC
UGUUUCCUGUGGGAUACCUGACUUCAGAUCAAG
AGA-’3

5’-AGUUGAUUGUGAAGCUGUUCGUGGA**GAUAUU**
UUUAAUCAGGUAGUUCCUCGGUGUCCUAGGUG
CCCAGCUGAUGAGCCACUUGCCAUCAUGAAGCC
AGAG-3’

NF-κB p65 blocking oligo

A.5’-TCCCACAGGAAACAGAAACCCCAGCTCCAGT
CAGAACTATTATCTTTTTGCA-3’
B.5’-GCTCATCAGCTGGGCACCTAGGACACCGAGG
AACTACCTGATTAAAAATATC-3’

Control oligo

5’-TGATTGAACAAGACCATCGTCCAGT-3’

B

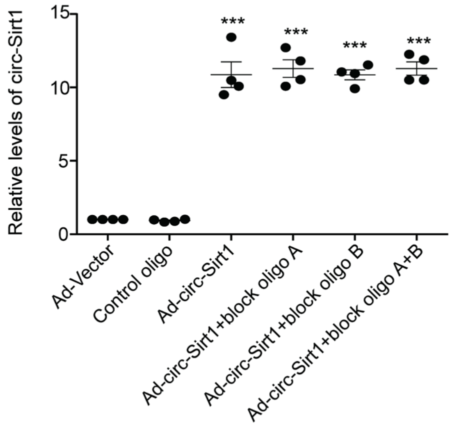

C

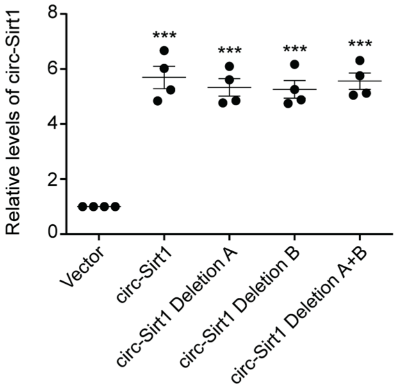

Supplementary Figure S7

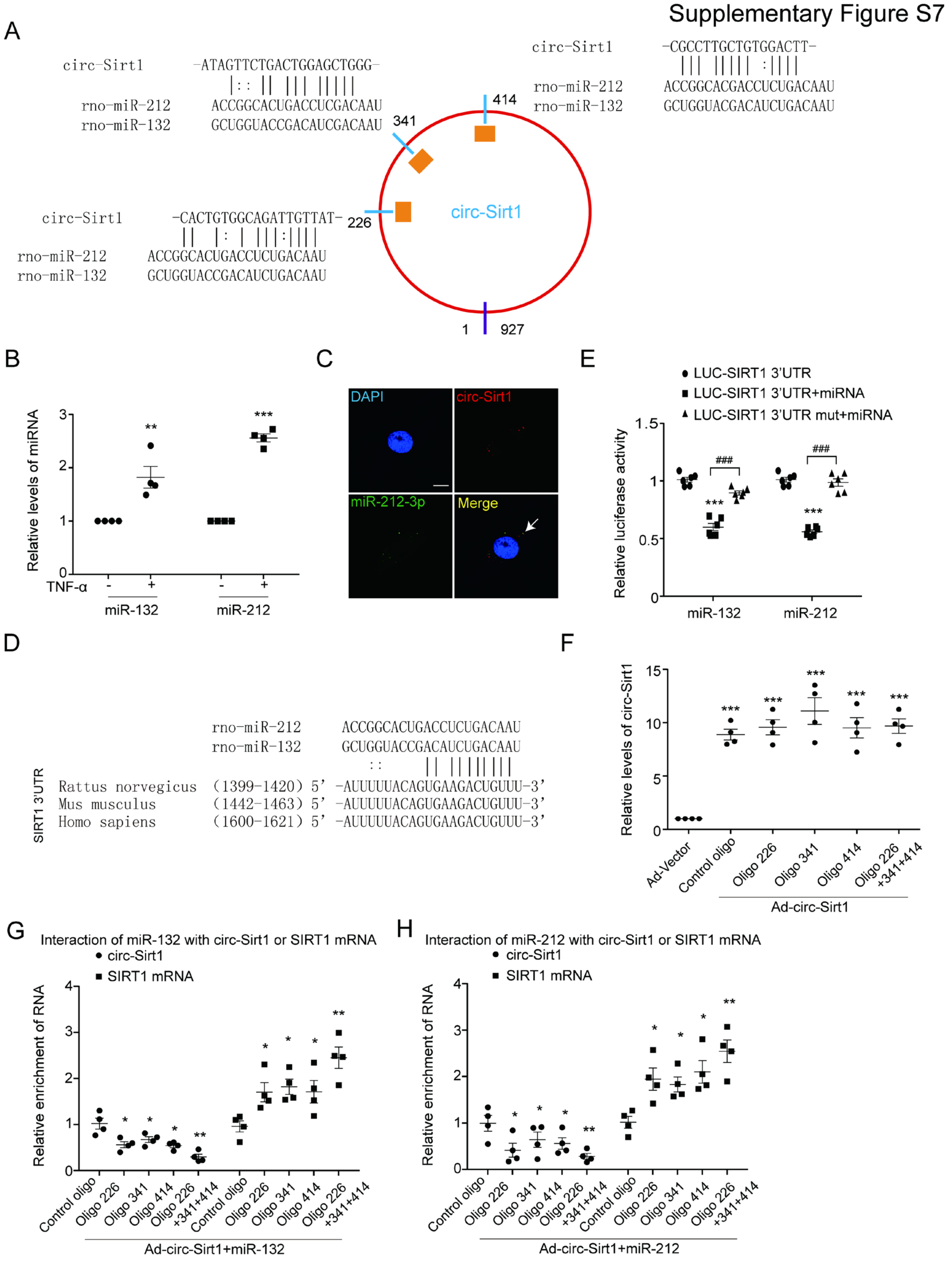

Supplementary Table S1. Clinical characteristics of patients for renal arteries collection

Clinical characteristics	Non-Atherosclerotic group (n=14)	Atherosclerotic group (n=11)	*P* value
**Demographics**			
Male sex, no. (%)	9 (64.3 %)	5 (45.5 %)	0.435†
Age (years)	53±3.72	62.27±1.93	0.053*
**Clinical Parameters**			
SBP, mmHg	121.64±1.75	158±5.68	˂ 0.0001*
DBP, mmHg	77.21±2.01	94.09±3.75	0.0003*
Heart rate, beats per minute	80.36±2.30	75.09±2.20	0.118*
**Comorbidities**			
Hypertension, no. (%)	0 (0)	11 (100 %)	NA
Type II diabetes mellitus, no. (%)	3 (21.4 %)	3 (27.3 %)	1†
**Laboratory Findings**			
Total cholesterol, mM	3.56±0.18	4.26±0.30	0.047*
Triglycerides, mM	1.23±0.13	1.74±0.22	0.044*
Creatinine, μM	69.23±7.80	81.31±6.98	0.273*

P value is calculated with t-test (*) or Fisher's exact test (†) to compare continuous variables (presented as mean±SEM) or categorical variables (presented as no. (%)), respectively.

Supplementary Table S2. Clinical characteristics of patients for peripheral blood collection

Clinical characteristics	Non-coronary stenosis group (n=20)	Coronary stenosis group (n=20)	*P* value
**Demographics**			
Male sex, no. (%)	15 (75 %)	14(70 %)	1†
Age (years)	52.6±3.38	61±2.83	0.064*
**Clinical Parameters**			
SBP, mmHg	127.25±1.99	144.1±4.7	0.003*
DBP, mmHg	77.65±1.67	82.75±1.73	0.041*
Heart rate, beats per minute	80.45±1.91	74.6±1.57	0.023*
degree of stenosis, no. (%)	0 (0)	87.8±1.95	NA
**Comorbidities**			
Hypertension, no. (%)	5 (25 %)	15 (75 %)	0.004†
Type II diabetes mellitus, no. (%)	1 (5 %)	6 (30 %)	0.091†
**Laboratory Findings**			
Total cholesterol, mM	4.13±0.21	4.72±0.09	0.016*
Triglycerides, mM	1.20±0.08	1.6±0.17	0.039*

P value is calculated with t-test (*) or Fisher's exact test (†) to compare continuous variables (presented as mean±SEM) or categorical variables (presented as no. (%)), respectively.

Supplementary Table S3. PCR primer sequence, siRNA sequence, and FISH probe sequence

Primer sequence		
circ-Sirt1 for rat	Forward	ACTAATTCCAAACAACCTCCTG
	Reverse	AACAATCTGCCACAGTGTCATA
complete circ-Sirt1 for rat	Forward	CTCTGTGTCACAAATTCATAGCTTTG
	Reverse	ATGGCTGGAACTGTCCGGGATATA
GAPDH mRNA for rat	Forward	ATCTTCCAGGAGCGAGATCCC
	Reverse	TGAGCCCTTCCACGATGCCAA
SIRT1 mRNA for rat	Forward	CGCCTTATCCTCTAGTTCCTGTG
	Reverse	TGCCTCTTGATCCCCTCCGTC
circ-Sirt1 for human	Forward	AGAGATTGTGTTTTTTGGTGAA
	Reverse	GAAGGTTATTTGGAATTAGTGC
GAPDH mRNA for human	Forward	ATCTTCCAGGAGCGAGATCCC
	Reverse	TGAGTCCTTCCACGATACCAA
VCAM-1 mRNA for rat	Forward	GGAGTCTGAACCCAAACAAAGG
	Reverse	GCAGTTCCCCGTTCTTTAGCT
ICAM-1 mRNA for rat	Forward	AAGAAGGACTGCTTGGGGAAC
	Reverse	TGGCGGTAATAGGTGTAAATGG
MCP-1 mRNA for rat	Forward	CACGCTTCTGGGCCTGTTGTT
	Reverse	GCCGACTCATTGGGATCATCTTG
VCAM-1 mRNA for human	Forward	GAAAAGTTCTTGTTTGCCGAG
	Reverse	GAAAGGTGCTGTAGATTCCCAT
ICAM-1 mRNA for human	Forward	GATTGTCATCATCACTGTGGTAG
	Reverse	GCCTGTTGTAGTCTGTATTTCTT
MCP-1 mRNA for human	Forward	TCTCTGCCGCCCTTCTGT
	Reverse	CGGAGTTTGGGTTTGCTTGT
siRNA sequence		
Negative control	Forward	UUCUCCGAACGUGUCACGUTT
	Reverse	ACGUGACACGUUCGGAGAATT
siRNA for circ-Sirt1	Forward	UCCAAACAACCUCCUGUUGTT
	Reverse	CAACAGGAGGUUGUUUGGATT
RNA pull down probe		
Oligo biotin		GTAGCACTAATTCCAAACAACCTCCTGTT GGC
circ-Sirt1 biotin probe		GCCAACAGGAGGTTGTTTGGAATTAGTG CTAC
miR-Con biotin		UUCUCCGAACGUGUCACGUTT

miR-132 biotin		CGACCATGGCTGTAGACTGTTA
miR-212 biotin		TGGCCGTGACTGGAGACTGTTA
Fish probe		
circ-Sirt1-Cy3		GCCAACAGGAGGTTGTTTGGAATTAGTGCTAC
miR-132-Fam		CGACCATGGCTGTAGACTGTTA
miR-212-Fam		TGGCCGTGACTGGAGACTGTTA
Chip primer		
NF-κB binding site	Forward	GTGGGTCCGTCCGTCTGTC
	Reverse	GGCTCGCCTCGGTACTTCT
Primers for plasmid constructs		
LUC-SIRT1 mRNA 3'-UTR-F		TTGGATCCATTATTTTTACAGTGAAGACTGTTTTCAGCTCTTTTTAGTCGACTT
LUC-SIRT1 mRNA 3'-UTR-R		TTGTCGACTAAAAAGAGCTGAAAACAGTCTTCACTGTAAAAATAATGGATCCTT
LUC-SIRT1 mRNA 3'-UTR-mut-F		TTGGATCCACAACCTCCTGTTGGCTGCAAAGATCATCACTAATGGCGTCGACTT
LUC-SIRT1 mRNA 3'-UTR-mut-R		TTGTCGACGCCATTAGTGATGATCTTTGCAGCCAACAGGAGGTTGTGGATCCTT
LUC-circ-Sirt1-F		TTGGATCCACAACCTCCTGTTGGCTG
LUC-circ-Sirt1-R		TTGTCGACTTGGAATTAGTGCTACTGGTC
pcDNA-circ-Sirt1-F		TTGATATCACAACCTCCTGTTGGCTG
pcDNA-circ-Sirt1-R		TTCCGCGGTTGGAATTAGTGCTACTGGTC
pcDNA-circ-GFP-F		TTGATATCGATCCGCCACAACATCGAG
pcDNA-circ-GFP-R		TTCCGCGGTTACTTGTACAGCTCGTCCATGC
